# In-Hover Aerodynamic Analysis of a Small Rotor with a Thin Circular-Arc Airfoil and a Convex Structure at Low Reynolds Number

**DOI:** 10.3390/mi14030540

**Published:** 2023-02-25

**Authors:** Yao Lei, Jie Wang, Yazhou Li, Qingjia Gao

**Affiliations:** 1 School of Mechanical Engineering and Automation, Fuzhou University, Fuzhou 350116, China; 2 Changchun Institute of Optics, Fine Mechanics and Physics, Chinese Academy of Sciences, No.88 Yingkou Road, Jingkai Area, Changchun 130000, China

**Keywords:** low Reynolds number, aerodynamics, small rotor, convex structure, hover

## Abstract

This study focused on the in-hover aerodynamics of a small rotor with a thin circular-arc airfoil and a convex structure at a low Reynolds number. The method combined computational fluid dynamics (CFD) with the blade element momentum theory (BEMT). The former was used for studying the two-dimensional parametric aerodynamics of the airfoil at a low Reynolds number and the latter was used for the prediction of the rotor’s hover performance. A novel thin circular-arc airfoil with a convex structure with a high aerodynamic performance, high structural strength, light weight and easy manufacturing process is presented in this paper. A convex curve on the upper surface was adopted to increase the thickness of the airfoil at partial chord, and a stiffener in the airfoil was installed to improve the structural strength of rotor span-wise. The aerodynamic performance of the airfoil was numerically simulated by the two-dimensional steady and incompressible Navier–Stokes equations. The in-hover performance of the rotor for small-scale vehicles was predicted by an improved version of the blade element momentum theory (BEMT). Finally, a carbon-fiber rotor with the presented airfoil was manufactured that had a diameter of 40 cm and a pitch of 6.2 inches. The analysis results were verified by experiments. It was shown that the maximum calculation errors were below 6%. The improved BEMT can be used in the analysis of in-hover micro-rotor aerodynamics at low Reynolds numbers.

## 1. Introduction

Recently, micro aerial vehicles (MAVs) have received great attention in the fields of civil and military applications such as structure inspection, search and rescue, delivering and remote sensing [1,2,3,4]. Due to the low rotational velocity and short chord length of rotor, MAVs always cruise over a Reynolds number (Re), ranging from 5000 to 300,000 [5,6]. This is much smaller than that of full-scale propellers, where Re ≥ 15,000,000. As Re decreases, laminar and viscous forces become dominant, resulting in a more complex boundary layer behavior as compared to large airfoils. Phenomena such as transition, separation and reattachment occur within a short chord-wise distance, which have a large effect on the pressure distribution and the airfoil’s performance [7,8,9,10,11]. In conditions with a low Re, the minimization of the airfoil′s shear drag is desired for full-scale design. This can be achieved by minimizing the regions along the airfoil with a turbulent boundary layer.

Schmitz [12] conducted an experimental investigation with a streamlined airfoil and thin, flat airfoils over a Re ranging from 42,000 to 420,000 with a low-turbulence wind tunnel. The results showed that by only reducing the laminar flow separation and air bubbles, the airfoils could demonstrate a good aerodynamic performance. In addition, Laitone and Lei [13,14] also conducted an experimental investigation to compare the aerodynamic characteristics and performance of a thin cambered-plate airfoil, a flat-plate airfoil and NACA0012 using a low-turbulence wind tunnel. The results showed that the thin cambered-plate airfoil generated the highest lifting force compared with the NACA0012. Xiao [15] studied the aerodynamic performance of Eppler61, Pfenninger048, a thin cambered-plate airfoil and a flat-plate airfoil. The results showed that the thin cambered-plate airfoil provided the highest lift and lift-to-drag (L/D) ratio.

As a result, these thin cambered-plate airfoils were put into use in multi-rotor MAVs at the University of Maryland, Stanford University and the Swiss Federal Institute of Technology. However, the structural strength and stiffness of these rotors were lower due to the small size of the rotors [16]. In actual applications, not only are the rotors easily damaged but the variation in their speed also results in poor aerodynamic performance. Paul [17] and Oscar [18] proved that thick airfoils can solve this problem, but thick airfoils (t/c > 6%) in a low Re regime tend to experience significant hysteresis in the lift and drag variation caused by the laminar–turbulent transition. In addition, a thick airfoil increases the mass of the rotors, which is not good for the flight time and payload capability.

Finding an optimal configuration of a rotor requires an intensive iterative process. For aerodynamic calculations, the current state-of-the art computational methods such as 3D computational fluid dynamics (CFD) [19], vortex modeling and lifting surface methods [20] that commonly used in full-scale design are still not mature enough to be efficiently used in small-scale applications. These methods require extensive experimental validation and vast computational resources, rendering them impractical for situations with limited resources and fast-paced design procedures. The blade element momentum theory (BEMT), combining the momentum and blade element theory, is a low-cost method that allows the estimation of the inflow distribution along the blade. Bohorquez [4] and Kunz [5] calculated the aerodynamics of a small rotor over a Re range from 15,000 to 60,000 using the BEMT method. In the process of the BEMT method, how to analyze the performance of the airfoil is crucial for particular designs [5,6,7,8,20].

In this paper, a novel airfoil configuration is introduced to maximize the aerodynamic performance of a rotor system at low Reynolds numbers. The aerodynamic performance of this airfoil was numerically simulated using the steady and incompressible Navier–Stokes equations. The in-hover performance of the rotor was predicted by an improved version of the BEMT. In addition, a carbon-fiber rotor with the presented airfoil was manufactured and tested in hovering conditions.

## 2. Material and Method

### 2.1. Airfoil

**Figure 1:**
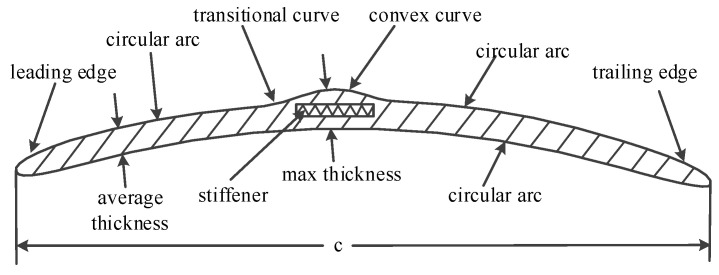
Schematic of thin circular-arc airfoil with convex structure.

Figure 1 shows the novel thin circular arc airfoil with a convex structure. It can be seen that a convex curve was added on the upper surface of the airfoil to provide space for stiffeners, which is different from the structure of traditional thin circular-arc airfoils. The airfoil consisted of a leading edge, a circular arc on the upper surface, a transitional curve, a convex curve, a trailing edge and a circular arc on the lower surface. These curves were connected one by one to form the airfoil. The convex curve on the upper surface was adopted to increase the thickness of the airfoil at partial chord. A stiffener was installed in this airfoil between the convex curve and the circular arc on the lower surface. This was to improve the structural strength of the rotor span-wise. An elliptical leading edge and trailing edge were used for easy fabrication.

Considering the fabrication process of the carbon fiber rotor, a layer of carbon fiber was required to be placed on the surface and lining cloth, and a unidirectional carbon fiber layer with a 0.2 mm thickness was used as a stiffener to acquire a high aerodynamic performance. The resulting thin circular-arc airfoil with a convex structure, a 2.5% uniform thickness and a 4.5% uniform camber served as the basis of the subsequent rotor airfoil. The size parameters of the airfoil are shown in Table 1.

### 2.2. Computation Scheme for Airfoil

One of the main features of the BEMT is the capability of incorporating geometric and operational rotor parameters, such as the blade platform, twist distribution, number of blades and collective pitch settings. However, the BEMT requires sectional airfoil characteristics in order to calculate the nonlinear axis-symmetric inflow. At larger scales, linear aerodynamics can be used to model an airfoil’s characteristics. Hence, a good estimate of an airfoil’s characteristics is key to achieving satisfactory results with this model.

To accurately predict the performance of small-scale rotors, it is necessary to obtain the sectional lift and drag coefficients of the airfoils used. For the research presented in this paper, the aerodynamic coefficients are obtained with CFD to calculate the baseline airfoil coefficients.

For steady and incompressible flow, we considered that the governing equations were the Navier–Stokes equations without gravity and body force items in a Cartesian tensor form:

(1) $$\frac{\partial u}{\partial x}+\frac{\partial v}{\partial y}+\frac{\partial w}{\partial z}=0$$

(2) $$\rho \left(u\frac{\partial u}{\partial x}\;+\;v\frac{\partial v}{\partial y}\right)\;=\mu \left(\frac{\partial^{2}u}{\partial x^{2}}\;+\;\frac{\partial^{2}u}{\partial y^{2}}\right)-\;\frac{\partial p}{\partial x}$$

(3) $$\rho \left(u\frac{\partial u}{\partial x}+v\frac{\partial v}{\partial y}\right)=\mu \left(\frac{\partial^{2}v}{\partial x^{2}}+\frac{\partial^{2}v}{\partial y^{2}}\right)-\frac{\partial p}{\partial y}$$

(4) $$\frac{\partial E}{\partial x}+\frac{\partial E}{\partial y}=W+Q$$

Equation (1) is the mass equation, and Equations (2) and (3) are the moment equations along the *x* and *y* axes, respectively. Equation (4) is the energy equation. *ρ* is the density of air, and *u* and *v* are the flow speed along the x and y axes, respectively. E is the total energy, and Q is the quantity of heat. The laminar flow viscous coefficient *µ* was given by the Sutherland equation. The viscous coefficient of turbulent flow was given by the turbulence model of Spalart–Allmaras [21]. The dissipation format was second order upwind, and the pressure and velocity were coupled through the SIMPLE algorithm. A C-type mesh generated by the elliptical method was applied to discretize the flow field of the airfoil. The external computational boundaries were set to be 20 chord lengths from the airfoil. The boundary conditions in this paper were as follows: (A) the upstream and lower boundary adopted the velocity–inlet boundary, i.e., the given value of the velocity; (B) the downstream and upper boundary adopted the pressure–outlet boundary and (C) the model surface adopted the wall boundary. The solution methods were second order with a standard pressure treatment. Different Reynolds numbers were realized by changing the inflow velocities.

### 2.3. Aerodynamic Performance of the Rotor

According to the in-hover momentum model [22], the thrust increment ∆*T* is given by the following equation:

(5) $$\Delta T=4\rho \pi v_{1}^{2}r\Delta r$$

where *r* is the dimensional radial coordinate (0 < *r* < D/2), *v*_1_ is the induced velocity of a section and ∆*r* is the segment the disk.

According to the in-hover blade element model [22], the element of thrust ∆*T* is given by the following equation:

(6) $$\Delta T=b\frac{\rho }{2}{\left(\Omega r\right)}^{2}a\left(\theta -\frac{v_{1}}{\Omega r}\right)c\Delta r$$

where *b* is the number of blades, Ω is the angular velocity, *a* is the slope of the lift curve, *θ* is the pitch angle of the blade element and *c* is the chord of the blade element. Then, the induced velocity is described by the following equation:

(7) $$v_{1}=\frac{-\frac{\Omega }{2}abc+\sqrt{{\left(\frac{\Omega }{2}abc\right)}^{2}+8\pi b\Omega^{2}ra\theta c}}{8\pi }$$

Based on the Mach number, the Reynolds number and the angle of attack, α, the lift and drag coefficients of cross sections C_l_ and C_d_ were defined with the CFD method, as presented in the section computation scheme for an airfoil.

If the lift and drag coefficients of a cross section are known, then the lift and drag forces that act on that segment, i.e., ∆*L* and ∆*D*, respectively, can be calculated. It is easily shown that the lift of a rotor is described by the following equation:

(8) $$T=b\int_{x_{0}R}^{BR}\frac{\Delta L}{\Delta r}dr=b\int_{x_{0}R}^{BR}\frac{\rho }{2}{\left(\Omega r\right)}^{2}a\left(\theta -\frac{v_{1}}{\Omega r}\right)cdr$$

where α is cross-sectional angle of attack.

(9) $$\alpha =\theta -\arctan\frac{v_{1}}{\Omega r}$$

For a single blade, the element of torque, ΔQ, is given by:

(10) $$\Delta Q=\Delta Lr\tan\Phi +\frac{\rho }{2}{\left(\Omega r\right)}^{2}C_{d}cr\Delta r$$

The rotor’s power requirement is given by:

(11) P=Ω×Q

The airflow conditions around a rotor can be characterized by the dimensionless Reynolds number on the rotor tip as follows:

(12) Retip=ρvbμ

where:

*ρ*—air density at the height of the rotor [Kg/m^3^].
*v*—rotor tip speed [m/s].
*b*—average chord length of the rotor [m].
*µ*—dynamic viscosity of air [Pa·s].

A figure of merit (FM) is used to compare the hovering efficiency of different rotors at the same disk loading. The BEMT results can provide the total thrust coefficient *C_T_* and the total power coefficient *C_P_* by the following equations:

(13) $$C_{T}=\frac{T}{\rho \pi R^{2}{\left(\Omega r\right)}^{2}}$$

(14) $$C_{P}=\frac{P}{\rho \pi R^{2}{\left(\Omega r\right)}^{3}}$$

where R is the blade radius. *FM* can be written as:

(15) $$FM=\frac{C_{T}^{3/2}/\sqrt{2}}{C_{P}}$$

## 3. Experiments

This section describes the experimental setup used for measuring the in-hover performance of the rotor. In order to experimentally measure the aerodynamic performance, a test apparatus was developed as follows.

**Figure 2:**
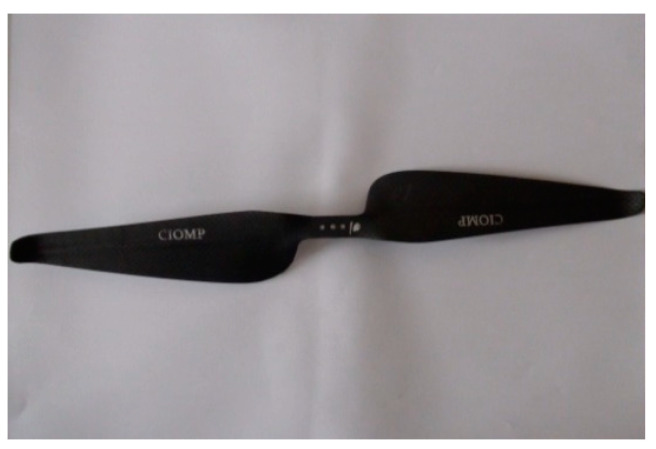
Manufactured rotor.

The experiments were conducted with a hovering system with a two-bladed carbon-fiber rotor. The rotor with unidirectional carbon-fiber fabrics used as stiffeners was manufactured based on a C5.5/4.5 airfoil and was 16 inches in diameter, 6.2 inches in pitch and 2.8 cm in chord at the 75% position. The mold was manufactured by CNC. The rotor, as shown in Figure 2, was made using a composite modulus in high-pressure conditions and had a weight of 15 g.

**Figure 3:**
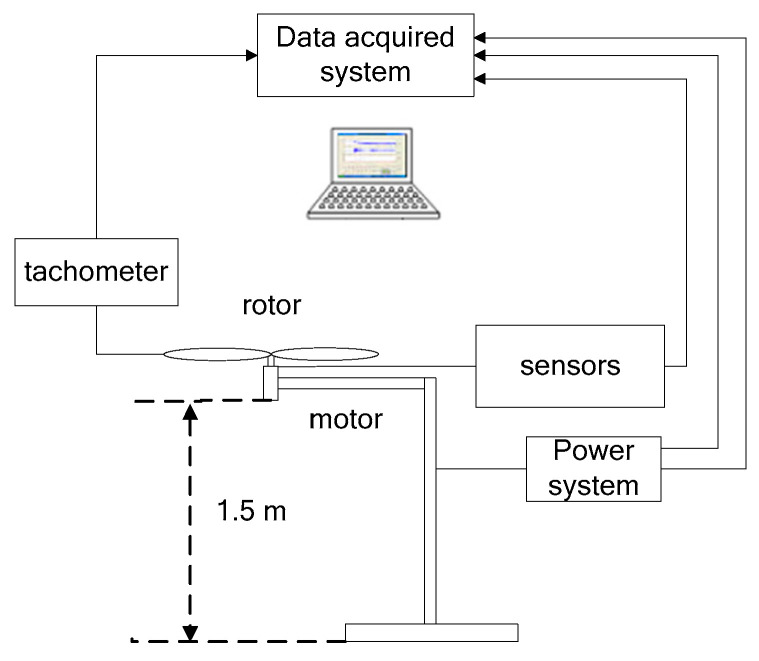
Schematic diagram of experimental setup.

A sketch of the test apparatus is shown in Figure 3. The rotor system was fixed inversely at a height of 1.5 m to avoid in-ground effects on the measurements. The rotor was driven by permanent-magnet brushless dc motor. Thrust was measured by a weighing sensor (type: CZL605, accuracy: 0.02% F.S), which was placed directly under the rotor shaft, while the rotational speed was measured by a tachometer (type: DT2234C, accuracy: ±(0.05% + 1 d)). Additionally, the voltage and current of the rotor system were also measured to obtain the power consumption using a digital multimeter (type: Agilent 34,401 A, accuracy: 6 1/2). In this setting, there was no extra hinge or bearing. So, the input signals, including the thrust, the rotational speed of the rotor, the current and the voltage were sent to the data acquisition system. The characteristics of the measurement system were as follows: (1) the rotor was mounted without ground effects and (2) no redundant parts were involved in the measurement system.

Thus, the rotor speed could be adjusted from 1300 r/min to 2300 r/min. The rotation speed, the electric current and voltage of the motor, and the voltage variation of the weighing sensor could be captured by the data acquisition system used in the experimental setup. The specific parameters of the experiment are shown in Table 2.

Error Analyses

Considering that the losses in the DC motor and the drift of the temperature were corrected by an electronic stability control (ESC) system, there were only two main error sources: the tachometer (rotational speed) and the thrust sensor (voltage variation). The error of a tachometer is related to the number of magnets in a motor. In this experiment, 24 magnets were used, and so there was an uncertainty of 1/24 of a revolution for every sample. The error generated by the thrust is proportional to the square of the rotational speed, and for a given rotational speed range, by applying the Kline–McClintock equation, the uncertainty of the thrust [23] is calculated as follows:

(16) $$\Delta C_{T}=\sqrt{{\left(\frac{C_{T}}{T}\Delta T\right)}^{2}+{\left(\frac{-2C_{T}}{\Omega }\Delta \Omega \right)}^{2}}$$

Finally, we have:

(17) $$\frac{\Delta C_{T}}{T}=\sqrt{{\left(\frac{\Delta T}{T}\right)}^{2}+4{\left(\frac{\Delta \Omega }{\Omega }\right)}^{2}}$$

According to the experimental data, the average uncertainty for thrust was about 1%.

## 4. Results and Discussion

**Figure 4:**
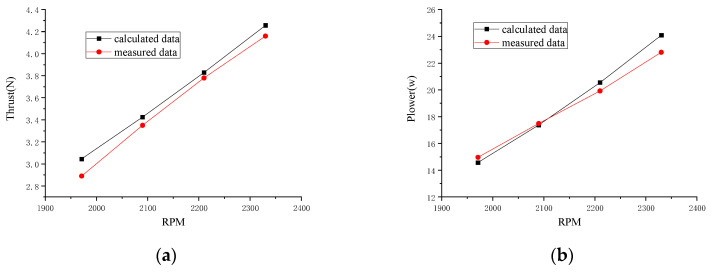
Comparisons of calculated data with measured data of hovering rotor. (**a**) Thrust distribution; (**b**) power consumption.

Figure 4 shows the computational and experimental data of the rotor’s in-hover aerodynamic performance. The efficiency was more than 10 g/w as the lift ranged from 150 g to 340 g and the power consumption ranged from 9 w to 31 w for 1300–2300 r/min. Figure 4a shows the distribution of the thrust with respect to the rotor speed acquired by experimentation and simulation. It can be seen that the results of the computation were higher than those of the experiment. The maximum and minimum errors were 5.4% at 1970 r/min and 1.3% at 2210 r/min, respectively. Figure 4b shows the distribution of the power loss with respect to the rotor speed. Figure 4 also shows that the results of the computation were lower than that of the experiment below 2090 r/min. The maximum and minimum errors were 2.6% at 1970 r/min and 0.6% at 2090 r/min, respectively. After 2090 r/min, the data of the computation were higher than those of the experiment with an error of 5.6% at 2330 r/min.

The total thrust and power consumption of the rotor were seen to increase fairly constantly with the speed, and an increase in the speed almost always resulted in a decrease in the power loading. The results showed that this method was accurate, concise and fast, and so it is suitable for rotor design.

**Figure 5:**
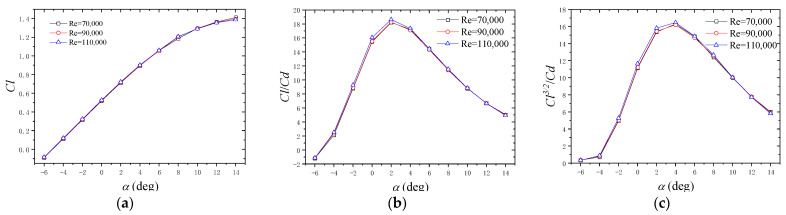
Aerodynamic performance of C5.5/4.5 airfoil. (**a**) Cl; (**b**) Cl/Cd; (**c**) Cl^3/2^/Cd.

Figure 5 shows the aerodynamic performance of the C5.5/4.5 airfoil at an angle of attack ranging from −6° to 14° for Re = 70,000, 90,000 and 110,000, respectively. Figure 5a shows the variation in the lift coefficient (Cl) with the angle of attack. We can see that Cl increased linearly from –6° to 6° and then increased non-linearly and reached a maximum of 1.41 at 14° for Re = 110,000. For the angle of attack ranging from −6° to 14°, Cl could be approximately expressed by a piecewise linear function, the interval of which was −6°–6°, 6°–10° and 10°–14°. For the angle of attack, the maximum and minimum slopes of the piecewise function were about 5.52 and 1.39, respectively, which were smaller than those of traditional lifting line slopes of about 5.73. Figure 5b shows the variation in Cl/Cd with the angle of attack. We can see that Cl/Cd firstly increased then subsequently decreased with the increase in the angle of attack, the maximum of which was 18.6 at an angle of attack of 2°. Cl/Cd increased as Re increased. Figure 5c shows the variation in Cl^3/2^/Cd with the angle of attack. We can see that Cl^3/2^/Cd firstly increased and then decreased with the increase in the angle of attack, the maximum of which was 16.5 at an angle of attack of 4°. Cl3/2/Cd increased as Re increased.

**Figure 6:**
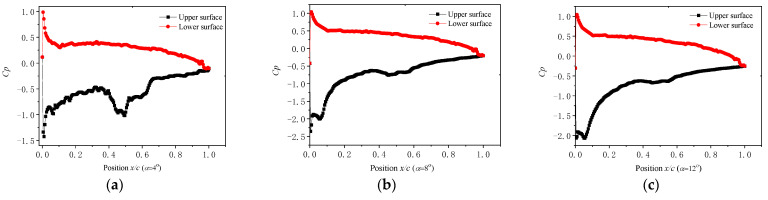
Pressure coefficient distribution line of presented airfoil surface at Re 70,000. (**a**) α = 4°; (**b**) α = 8°; (**c**) α = 12°.

Figure 6 shows the pressure coefficient distributions of the C5.5/4.5 surface at Re = 70,000. As shown in Figure 6a, at an angle of attack of 4°, the pressure coefficient on the upper surface gradually rose at the beginning and then decreased at about 32% of the chord, where the minimum was at 50% of the chord. Consequently, a positive pressure gradient (dp/dx < 0) area was formed. For the 50% of the chord length, the pressure coefficient increased gradually and then formed a large inverse pressure gradient area, which was mitigated after 65% of the chord length. The pressure coefficient on the lower surface decreased gradually from the leading edge to the trailing edge. The pressure coefficient turned to be negative at 99% of the chord. As a result, the convex curve on the upper surface was beneficial for improving lift coefficient at an angle of attack of 4°. As shown in Figure 6b, the pressure coefficient distribution on the upper surface was similar to that at an angle of attack of 4°. However, the positive pressure gradient area became smaller, and the adverse pressure gradient did not change significantly. The pressure coefficient on the lower surface turned negative at 97% of the chord. As shown in Figure 6c, the positive and adverse pressure gradient on the upper surface became more and more non-significant. The pressure coefficient distribution line on the lower surface was similar to that at an angle of attack of 4°. A negative pressure coefficient occurred at 94% of the chord.

**Figure 7:**
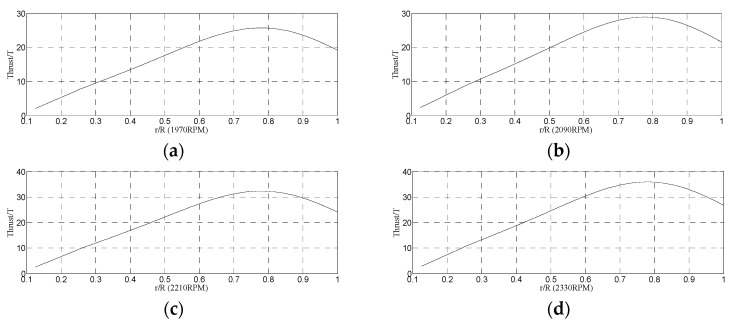
The thrust distribution at (**a**) 1970 r/min, (**b**) 2090 r/min, (**c**) 2210 r/min and (**d**) 2330 r/min.

Figure 7 shows the thrust distribution at 1970 r/min, 2090 r/min, 2210 r/min and 2330 r/min. It can be seen that the thrust increased linearly as the position was less than 0.6 R and reached a maximum at 0.75 R.

**Figure 8:**
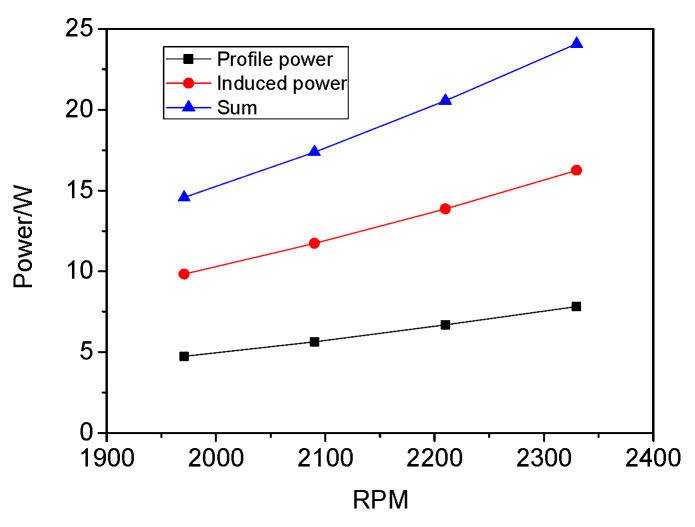
Power consumption.

The momentum theory gives the induced power consumption of an ideal hovering rotor, i.e., $C_{pi}=C_{T}^{3/2}/\sqrt{2}$. A real rotor generates power consumption from other sources as well, in particular the profile power loss due to the drag of the blades in a viscous fluid. In addition, there is power consumption due to uniformity in the inflow, swirl in the wake and swirl in the tip. Compared with the experimental data, these power losses were relatively small. As shown in Figure 8, the induced power loss had a percentage of 67.5% of the total power losses, and the profile power loss had a percentage of 32.5%.

## 5. Simulations

**Figure 9:**
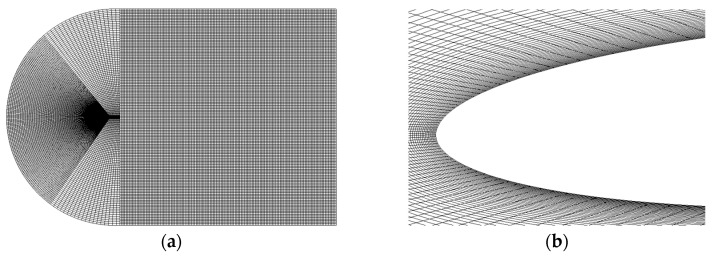
Mesh distribution. (**a**) Mesh domain; (**b**) mesh on the leading edge of airfoil.

In this section, numerical simulations for the novel thin circular-arc airfoil with a convex structure were carried out to visualize the results. The flow domain was fully structured and consisted of 100,000 quadrilateral cells, as presented in Figure 9a. The mesh became denser around the airfoil and at the leading and trailing edges, as shown in Figure 9b.

**Figure 10:**
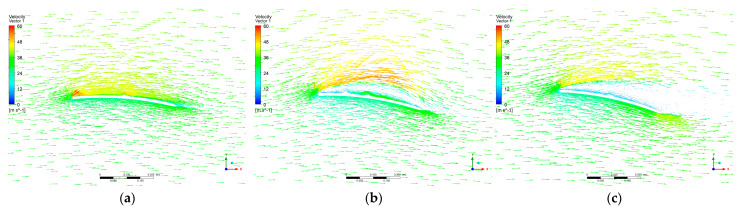
Velocity vector of proposed airfoil at Re of 600,000. (**a**) Angle of attack of 4°; (**b**) angle of attack of 8°; (**c**) angle of attack of 12°.

Figure 10 shows the velocity vector of the airfoil for a Reynolds number of 60,000. As shown in Figure 10a, two high-speed regions appeared at the leading edge of the upper surface for 4°, where the Bernoulli equation showed that the low pressure on the upper surface was relatively small. The boundary layer flow between the leading edge and the raised curve of the upper surface of the airfoil tended to be laminar, but the boundary layer flow between the raised curve and the trailing edge already showed a laminar separation. As can be seen in Figure 10b, the laminar separation increased at the trailing edge of the upper surface and decreased in the high-speed region at 8°. Vortices appeared on the upper surface of the rotor blade. In addition, as shown in Figure 10c, there were still two significant laminar separations at 12°, but the intensity of the vortices on the upper surface was decreased compared to 8°.

**Figure 11:**
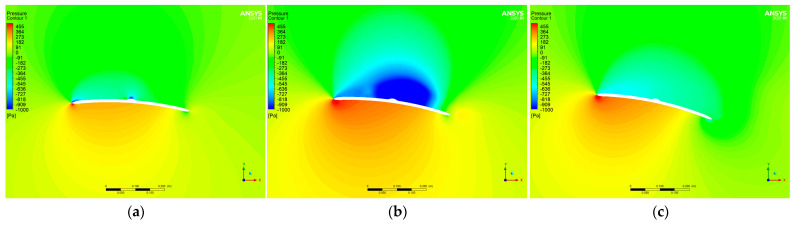
Pressure of proposed airfoil at Re of 60,000. (**a**) Angle of attack of 4°; (**b**) angle of attack of 8°; (**c**) angle of attack of 12°.

Figure 11 shows the pressure distribution of the airfoil for Re of 60,000. At an angle of attack of 4°, it can be seen that, as expected, there was a strong low pressure on the leading edge. At an angle of attack of 8°, there was a large area of low pressure between the protruding curve of the airfoil and the trailing edge. This was due to the creation of vortices, as can be seen in Figure 8. The protruding part of the airfoil was, to some extent, an obstacle to the creation of vortices. At an angle of attack of 12°, no significant change in pressure could be seen in the projecting curve of the airfoil. Therefore, at an angle of attack of 12°, the protrusion had almost no effect on the aerodynamic performance of the airfoil.

**Figure 12:**
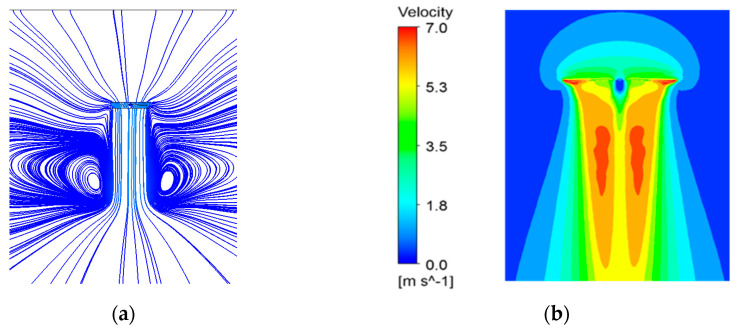
Downwash of the rotor. (**a**) Streamline; (**b**) velocity.

According to the momentum theory, downwash flow affects a rotor’s thrust. The streamline and velocity distributions of the downwash for a working mode of 2200 rpm are shown in Figure 12.

Figure 12 shows that a pair of vortices formed below the rotor where the velocity of the airflow was increased by the acceleration of the rotor. In addition, there was a tendency for the high-velocity area of the airflow to move towards the rotor center. Eventually, the velocity of the downwash flow reached its maximum and then gradually decreased again.

**Figure 13:**
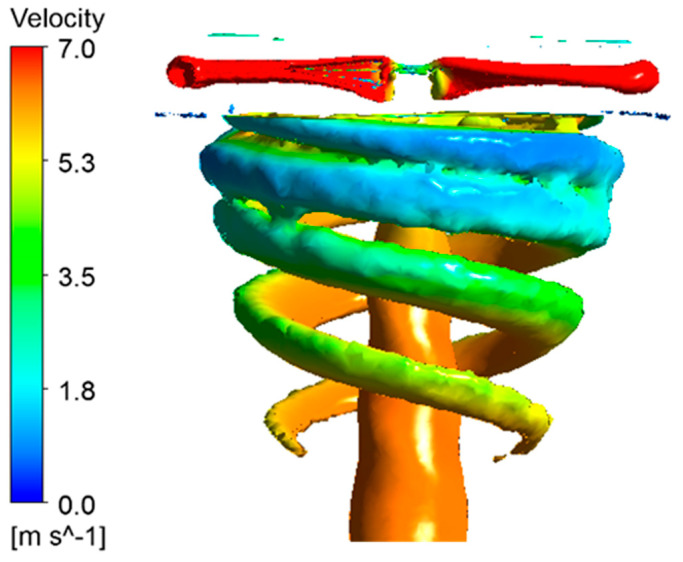
Free wake of the rotor.

Figure 13 shows the captured free wake of the rotor at 2200 RPM.

It can be seen that the numerical simulations captured the creation and development of the free wake of the rotor, which gradually developed downwards from the tip of the rotor, starting tightly and then gradually thinning and contracting, which can be used as guidelines for future simulations of tip vortex flow fields.

## 6. Conclusions

In this paper, the aerodynamic performance of a circular=arc airfoil with a convex curve was studied with experiments and simulations. The profile of the proposed airfoil was proven, achieving great aerodynamic performance at low Re.

(1) For the C5.5/4.5 airfoil, Cl/Cd reached a maximum of 18.6 at an angle of attack of 2°, and Cl3/2/Cd reached a maximum of 16.5 at an angle of attack of 4° with an Re of 70,000, 90,000 and 110,000, respectively. Therefore, an angle of attack from 2° to 4° is more suitable for rotors to achieve a better aerodynamic performance in future design.

(2) The thrust distribution and power loss were important for the optimal design of the rotor. The thrust element increased linearly as the position was less than 0.6 R and then increased non-linearly increases and reached a maximum at 0.75 R. In addition, the power loss mainly consisted of the profile power loss and induced power loss at low Re. The induced power loss had a greater percentage of 67.5%.

(3) The optimal rotor with unidirectional carbon fiber fabrics used as stiffeners was manufactured based on a C5.5/4.5 airfoil and 16 inches in diameter, 6.2 inches in pitch and 2.8 cm in chord at the 75% position. The aerodynamic performance of the rotor calculated by an improved version of the BEMT was compared with the experimental results, and the calculation errors of the lift and power loss were both below 6%. The rotor profile can thus be applied in multi-rotor MAVs.

(4) Finally, the convex curve on upper surface was beneficial for improving the lift coefficient at an angle of attack of 4°, which showed good agreement in terms of the aerodynamic model for both the simulations and measurements.

Future works could include obtaining an aerodynamic database of circular-arc airfoils, constructing a model of a rotor together with a motor and the interaction of multiple rotors on aerodynamic performance.

## Figures and Tables

**Table 1:** Design parameters of the airfoil.

Parameter	Value
Max. thickness	4.3%
Max. camber	5.5%
Average thickness	2.5%
Average camber	4.5%

**Table 2:** Specific parameters of the experiment.

Parameter	Value
Reynolds number range	70,000–110,000
RPM range	1300–2300 (r/min)
Air density	1.28 (kg/m^3^)
Dynamic viscosity	1.82 × 10^–5^ (kg/ms)
Rotor angle of attack	−6–14 (deg)

## Data Availability

All data are already included in the manuscript.
